# COVID-19 lockdowns and working women’s mental health: Does motherhood and size of workplace matter? A comparative analysis using understanding society

**DOI:** 10.1016/j.socscimed.2023.116418

**Published:** 2023-11-16

**Authors:** Jessica Wilson, Evangelia Demou, Theocharis Kromydas

**Affiliations:** a College of Social Science, University of Glasgow, Glasgow, United Kingdom; b MRC/CSO Social and Public Health Sciences Unit, School of Health and Wellbeing, University of Glasgow, Glasgow, United Kingdom

**Keywords:** COVID-19, Mental health, Mother, Employment, Lockdown, SME

## Abstract

The COVID-19 pandemic had detrimental and unequal repercussions on mental health. To date there is little evidence exploring how motherhood and workplace size moderates this relationship for working women. This study aimed to estimate changes in working women’s mental health at the start of each UK lockdown and estimate the effect of motherhood and workplace size on mental health. We used Understanding Society data from women in paid employment, who participated in at least: one pre-COVID-19 Wave (9 or 10/11) and one COVID-19 lockdown wave (Lockdown 1: April 2020, Lockdown 2: November 2020, Lockdown 3: January 2021). Primary outcome was probable psychological distress (i.e., score≥4 in the General Health Questionnaire-12 (GHQ-12)). In Model 1, exposure was motherhood (binary), interacting with a variable that split time in the pre-pandemic and lockdown periods. In Model 2, workplace size (Micro:1–24, Medium: 25–199, Large: More than 200 employees) was added as an exposure (3-way interaction) to investigate moderation effects. We fitted mixed–effects logistic regression models, adjusting for age, ethnicity, UK country of residence, cohabitation, educational qualifications, working hours, furlough, subjective financial difficulty and previous health condition. In the adjusted Model 1, pre-pandemic, odds of GHQ-12 caseness were lower for mothers compared to non-mothers (OR:0.89 95%CI:0.77,1.03). However post-pandemic compared to pre-pandemic, odds for mothers were higher than non-mothers, especially during lockdown 3 (Non-mothers: OR:1.93 95%CI:1.69,2.20; Mothers: OR:2.87 95%CI:2.36,3.49). In Model 2, workplace size did not modify the relationship. Pre-pandemic, there was no difference in the odds of GHQ-12 caseness by workplace size; however, the differences observed in Lockdown 3 between non-mothers and mothers, are mainly attributed to differences in medium-sized enterprises (Non-mothers: OR:1.95 95%CI:1.53,2.48; Mothers: OR:3.56 95%CI:2.54,4.99). Future policies should be designed to facilitate the working lives of mothers, but especially for medium-sized enterprises as extreme uncertainty appears to affect these employees more.

## 1 Introduction

The COVID-19 pandemic has precipitated numerous unforeseen implications and adversely affected both physical and mental health (Almeida et al., 2020; Das et al., 2021; Galanti et al., 2021; Hamouche, 2020; Kromydas et al., 2022; Michael et al., 2022; Mutambudzi et al., 2020; C. L. Niedzwiedz et al., 2021b; C. L. Niedzwiedz et al., 2020). In an effort to successfully contain the spread of the virus, governments implemented non-pharmaceutical interventions known as lockdowns (UK Government, 2020a). These interventions consisted of social distancing measures, including physical distancing, remote work, restricted business hours, school closures, and gathering limits (Ferguson et al., 2022; Le et al., 2020; Meyerowitz-Katz et al., 2021). In the United Kingdom (UK), three national lockdowns were enforced that varied in rules, regulations and length of enforcement (Baker et al., 2021; Institute for Government Analysis, 2021). In the UK, the 1st and 3rd lockdowns consisted of mainly similar rules, while the 2nd lockdown was more lenient in comparison (Fig. A1) (Baker et al., 2021; UK Government, 2020a, 2020b).

Though non-pharmaceutical interventions against COVID-19 have been effective in reducing infectious disease transmission, they have also adversely impacted psychological wellbeing (Jacobson et al., 2020; Kromydas et al., 2022; Le et al., 2020; Marroquin et al., 2020; C. L. Niedzwiedz et al., 2021b; C. L. Niedzwiedz et al., 2020; Patel et al., 2022; Pieh et al., 2020). Many countries, including the UK, reported increased rates of anxiety, depression, and drug and alcohol use, among other indicators of mental illness, following the onset of the pandemic (Das et al., 2021; Hampshire et al., 2021; Nelson and Kaminsky, 2020; C. L. Niedzwiedz et al., 2021b; Patel et al., 2022). Unequal trends in pandemic-related deterioration of mental health have been reported, with particular populations including women, parents, and certain employee groups more adversely affected than others (Almeida et al., 2020; Arntz et al., 2022; Kromydas et al., 2022; Michael et al., 2022; Mutambudzi et al., 2020; C. L. Niedzwiedz et al., 2021b; Patel et al., 2022; Pieh et al., 2020; Pierce et al., 2020; Xiao et al., 2021). Research published thus far has either tended to combine lockdown periods (most common approach), categorised lockdown periods by restriction levels or analysed single lockdown periods only (Cheng et al., 2021; Kallitsoglou and Topalli, 2021; Michael et al., 2022; Mutambudzi et al., 2020; C. L. Niedzwiedz et al., 2021b; Pieh et al., 2020). Such approaches, however, overlook the impact of potential variations in restrictions enforced during each lockdown and as a result, the evolution of psychological health throughout the course of the pandemic is under-investigated. Additionally, employment, family factors, and differences by UK lockdowns regarding working women’s mental health status is still scarce (Garner, 2021; Grekou and Lu, 2021; Michael et al., 2022; Nishikido et al., 2023). Evaluating how the initial stages of each introduction of COVID-19 public health measures -i.e., lockdowns-impacted mental health, is of particular importance. These are the time periods when strain, uncertainties and demands on working women, especially mothers, were greatest (Kallitsoglou and Topalli, 2021). For instance, a study conducted recently demonstrated that couples with children incurred a higher risk of psychological distress compared to couples with no children during UK lockdowns (Michael et al., 2022). These issues further highlight the need to address the increased prevalence of psychological distress and widening health inequalities proceeding the pandemic (Paremoer et al., 2021; Patel et al., 2022).

The workplace and one’s job can greatly influence health and well-being (Waddell and Burton, 2006). The prevalence and implementation of proactive workplace policies, practices and interventions to support employees, improve and sustain employee health is not directly a function of workplace size. However, workplace size is associated with a range of organisational and operational characteristics. Micro, small and medium sized enterprises (SMEs) have distinct characteristics that differentiate them form large employers; for instance, Lindeque et al. (2022) report how SMEs are generally independent, multi-tasking, based on personal relationships and informality, and are actively managed by the owners. The workforce make-up of workplaces by size has also been changing, with three-fifths of employment in the UK private sector being in SMEs (BEIS, 2022). The type of work also varies by workplace size; wholesale and retail trade being the industrial sector with the highest share in SMEs, whereas in large enterprises manufacturing has the highest share, followed by administrative and support services and wholesale and retail (BEIS, 2022). Furthermore, resources available to employees and formal or informal occupational health provision is often limited and or not present in SMEs (Champoux and Brun, 2003). In the UK, only 18% of small employers provide occupational health (OH) services compared to 92% of large employers (Tu et al., 2019). Provision of proactive health promotion interventions also varies by employer size, and tends to be lower in smaller workplaces (Burge et al., 2023). Financial constraints, along with the lack of knowledge and support to implement services are key barriers for smaller workplaces (Burge et al., 2023).

The differences in employment type, support, organisational structure between workplaces of different size, also results in differences in risks, demands and rewards that can differentially impact on health and wellbeing. The theoretical framework that underlies this is the Job-Demands and Resources (JD-R) model, developed in 2006 (Bakker and Demerouti, 2007). The basic principle of the JD-R is that when job demands are high and job resources are low, stress and burnout increase (Bakker and Demerouti, 2007). In cases when demands are high, resources to compensate and mitigate these effects become increasingly more valuable (Galanakis and Tsitouri, 2022). As stated by Galanakis and Tsitouri (2022) the JD-R is a “holistic model that can be applied to a variety of occupational settings, regardless of the specific demands and resources implicated”. Earlier research found that role conflict workload, and low support were the ‘job demands’ most detrimental to employee wellbeing, whereas most beneficial ‘resources’ were support from others, performance feedback, job control and autonomy among others (Galanakis and Tsitouri, 2022). The aforementioned, job demands and resources, differ significantly by workplace size, mainly due to the culture, organisational management and resources available by workplace size (Llanos-Contreras et al., 2023; Siegrist, 2023; A. Wagner et al., 2022).

While, the JD-R model has proven to be reliable and used in the COVID-19 pandemic (Llanos-Contreras et al., 2023; Mohammed et al., 2022; Scheel et al., 2022), Demerouti and Bakker (2022) have now proposed that during periods of crisis job characteristics alone may not sufficiently explain employee health. Work and non-work factors, such as non-work demands need to be considered (Demerouti and Bakker, 2022), and to our knowledge this has not been considered to date for the assessment of employees’ mental health across workplace size.

Building on the plethora of research that shows that women’s mental health was more adversely affected compared to men, this study focuses on working women only, and seeks to fill an important research gap. We investigate whether and how much UK working mothers’ psychological well-being changed and how this compared to working non-mothers during the initial stage of each of the three COVID-19 lockdowns. Furthermore, we investigate how workplace size moderates any potential change in mental wellbeing, as the demands, resources and support and flexibility offered from employers is often related to employer size (Bradshaw et al., 2001b; Behdin Nowrouzi et al., 2016b). We used a novel approach comparing mental health in the first month of each of the three UK lockdowns to a pre-COVID-19 pandemic period spanning from 2017 until March 2020. Using the theoretical framework of the job demands-resources (JD-R) model that underlies this research, we hypothesise that working women’s mental health is likely to be different by motherhood, periods of lockdown and size of workplace. The specific research questions (RQ) this study aimed to answer were:

**RQ1**. How did working mothers’ mental health change compared to working non-mothers with the introduction of each UK lockdown compared to pre-pandemic levels?

**RQ2**. Comparing each UK lockdown separately with the pre-pandemic period, does workplace size moderate potential mental health differences between working mothers and non-mothers?

## 2 Data

We used data from Understanding Society (USoc), a nationally representative, open-cohort household panel study with a cluster-stratified probability sample consisting of UK households that are interviewed annually (University of Essex, 2021). For the comparative analysis between pre-pandemic period and each lockdown, we used data from participants interviewed in at least: one pre-COVID-19 Wave, and the COVID-19 survey that corresponded to the first month of each lockdown (lockdown 1: April 2020, lockdown 2: November 2020, lockdown 3: January 2021), to capture mental health at the initial stages of each lockdown. Fig. A1 summarises the basic restrictions in each of the three lockdown periods.

The population of interest was working women aged 16 years or older residing in the United Kingdom. The age range in our final analytical sample was 18–84 with just 3.7% between 65 and 84 years old. Men, proxy respondents, and those not in work were excluded.

Working women were defined as women who self-reported being employed and/or self-employed in each survey wave. More than a third of respondents in our analytical sample participated in all 6 waves (38%), while more than a half missed just one wave (55.6%). Table A1 details the times respondents participated in our analytical sample.

## 3 Empirical strategy

The outcome assessed was probable psychological distress using the General Health Questionnaire-12 (GHQ-12). This was collected at each wave/time point of interest. GHQ-12 is a validated screening tool for psychological distress, specifically assessing the risk of anxiety and depression (Davillas and Jones, 2021; Goldberg et al., 1988; C. Niedz-wiedz et al., 2021a; University of Essex, 2021). For the purposes of this study, participants who scored ≥4 on the GHQ-12 were coded as likely experiencing anxiety/or depression (i.e., GHQ-12 ‘caseness’) (Kromydas et al., 2022; C. L. Niedzwiedz et al., 2021b). Sensitivity analysis was conducted by using a one-point lower cut-off point (GHQ-12 score ≥3).

The exposures of interest were time-period (pre-pandemic/lockdowns 1, 2, & 3), motherhood (mother/non-mother), and size of workplace (micro, medium and large companies). Time period was a categorical variable with four values; the reference category was pre-COVID-19 (Waves 9, 10 and 11 spanning from 2017 to March 2020) and three values represented the COVID-19 surveys conducted during the first stages-approximately a month-of each UK lockdown. Lockdown 1 is therefore, represented by COVID-19 survey 1 (Mar-Apr, 2020), lockdown 2 by survey 6 (Nov-Dec 2020), and lockdown 3 by survey 7 (Jan-Feb 2021). Motherhood was measured using a binary variable representing working women as being either a mother or not. Working mothers were defined as women who reported having children under 16 years old in the household regardless of whether that child was a step, adopted, or biological child (Cheng et al., 2021). Motherhood was further stratified by the size of workplace participants were employed in. We recoded the original USoc variable for workplace size into 3 major categories: micro: 1–24 employees (including the self-employed), medium: 25–199 employees and large: over 200 employees.

We adjusted for potential demographic, family, economic and health related confounders that may differentially affect change in mental health across our exposure groups. Demographic confounders included: age as a continuous variable, age square, ethnicity (white/non-white), UK country of residence (England, Scotland, Northern Ireland, Wales), and level of educational qualification (none, intermediate, higher). The family status confounder used was living situation (i.e., living with a partner or not). Financial situation was represented by a variable capturing the subjective judgement of a respondent’s financial situation. The original variable values were five (1: Finding it very difficult to cope with present income, 2: Finding it quite difficult to cope with present income, 3: Just about getting by, 4: Doing alright, 5: Living comfortably). We converted this into a binary format (0: Good, 1: Bad), where ‘Good’ is represented by values 4 and 5; and ‘Bad’ by values 1,2 and 3 of the original variable. Working hours are captured by a categorical variable (0: working 1-15 h/week, 1:16-35 ;h/week and 3: More than 36 h/week). Baseline health condition was categorised by a binary variable that captured long-standing illness or impairment (0: No, 1: Yes). We carried data forward and backwards for educational qualifications, financial situation, and baseline health condition to boost observations in our sample (Appendix; Section: Methods). The sampling weights Understanding Society provide were constructed taking into account both genders; therefore, these weights could not be applied to a sub-sample of only one gender. Instead, we accounted for outcome missingness calculating relevant Inverse Probability Weights (IPW) (Appendix; Section: Weighting).

Descriptive statistics were employed to analyse the characteristics of the survey respondents. Two series of mixed–effects binary logistic regression models with a log-linear link function were then fitted to assess the odds of GHQ-12 caseness by exposure group (time period interacted with motherhood (Model 1) and then, additionally with workplace size (Model 2). We accounted for repeated observations over time on the individual level and adjusted for serial correlation of observations within individual values using the cluster sandwich estimator, which allows for intragroup correlation in standard errors. We estimated both unadjusted and adjusted results for both models. Odds ratios (OR) as well as marginal means (i.e., probability of GHQ-12 caseness for all groups) and average marginal effects (AME), (i.e., the average probability difference between mothers and non-mothers, pre and during pandemic as well as between workplaces of different sizes) were used to estimate both relative and additive changes in psychological distress in the first stages of each UK lockdown compared to the pre-COVID-19 period (Kromydas et al., 2022). The regression equation, for adjusted Models 1 and 2 is as following:

$$GHQcase{\;}_{iw}=a_{0}+\gamma X_{iw}+\theta Z_{iw}+\mu_{i}+\pi_{w}+e_{iw}$$

where i represents individuals, w represents waves, α_0_ is the intercept, γ represents the vector of our exposure groups as explained above, and θ represents a vector of all our additional covariates. The letters μ and π represent individual and wave fixed-effects respectively, while ε is the error term that varies randomly across waves. When ORs are estimated, interaction effects are calculated in a multiplicative manner (Model 1: logit (P{GHQ_case_ = 1})^time period*motherhood^ and Model 2: logit (P {GHQ_case_ = 1}^time period*motherhood*workplace size^), whereas marginal means represent additive effects ((P{GHQ_case_ = 1})time period + motherhood and Model 2: (P{GHQ^case^ = 1}_time period + motherhood + workplace size_).

In Model 1, exposure was being a working mother (binary), interacting with a categorical variable that splits time in the pre-pandemic and the three lockdown periods as distinct values. In Model 2, workplace size was added as an additional exposure in Model 1 (3-way interaction) to investigate moderation effects.

## 4 Results

The total analytical sample consisted of 26,077 observations (pre-pandemic: 14,411, lockdown 1: 5,026, lockdown 2: 3,606, and lockdown 3: 3034) across 5540 individuals (Fig. A2). As shown in Table 1, for most confounders, there were no substantial differences between the pre-COVID-19 and lockdown periods. However, working women in lockdowns 2 and 3 appear older compared to lockdown 1 and the pre-COVID period (pre-COVID: 44.52 ± 12.21 years:Wave 9: 43.97 ± 11.90, Wave 10: 44.53 ± 12.24, Wave 11: 45.02 ± 12.45) lockdown 1: 45.37 ± 12.43 years, lockdown 2: 47.10 ± 12.20 years, lockdown 3: 47.51 ± 12.15 years). For ethnicity, the percentage of non-white participants ranges from 9 to 10.5% across all waves used in our analysis. The majority reported living in England (81%–82%) and 58%–59% of all participants had a higher level of education. About 1/3 of the sample population were working mothers (29%-35%) and almost three-quarters lived with a partner or spouse (69%–71%). Most working women worked at a micro or medium-sized enterprise (68%–70%). Working hours dropped in all lockdowns - especially in lockdown 1 - compared to pre-COVID periods (Table 1). Overall, most participants (61.% to 88%) worked more than 16 h/week. Around 22%–26.5% reported being in a ‘bad’ financial situation (i.e., finding it very difficult, difficult to cope or just about getting by) while 27%–28% disclosed a long-standing illness or impairment.

**Fig. 1:**
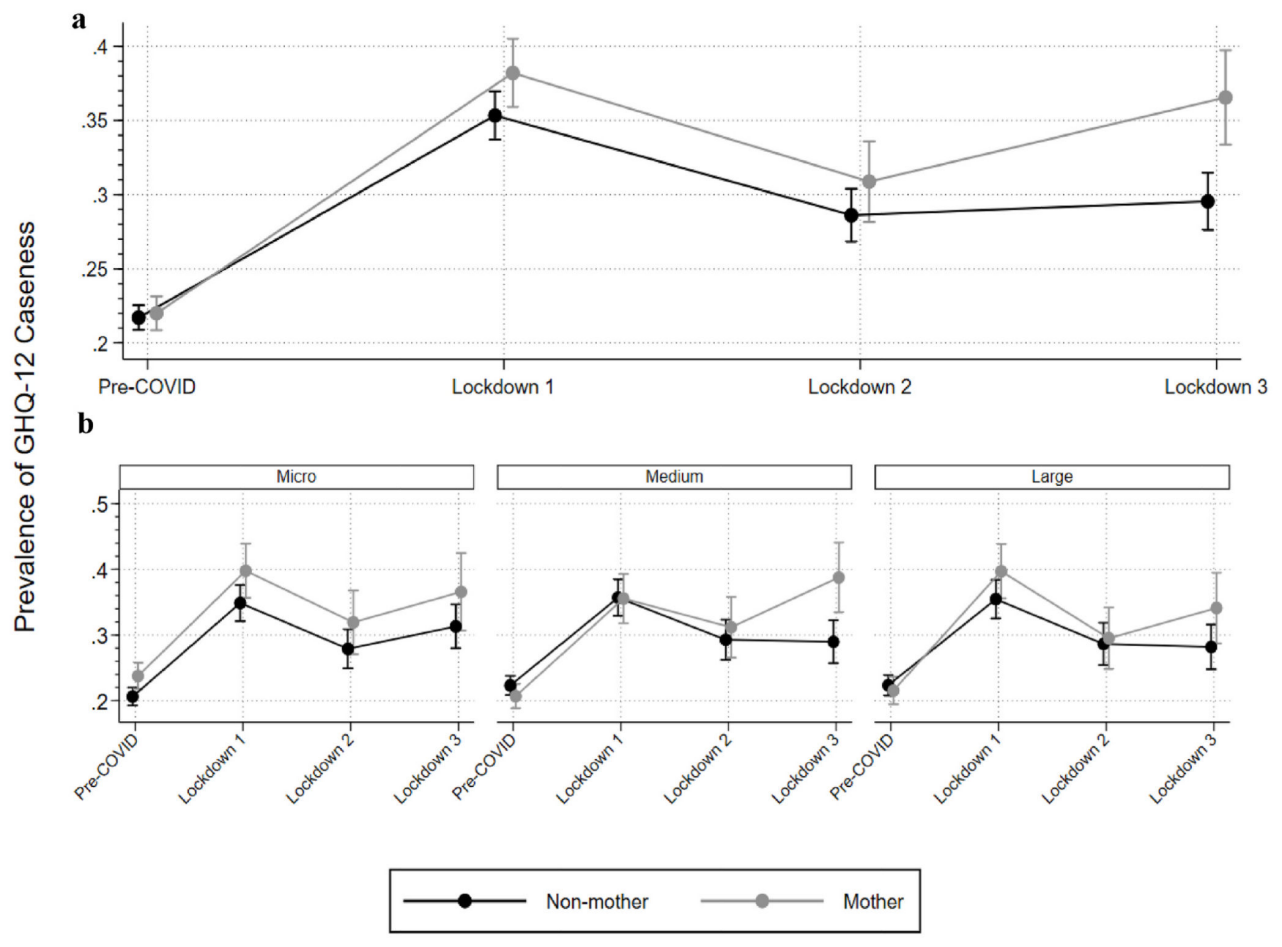
Prevalence of GHQ-12 caseness by motherhood and time period (a-top graph) and by motherhood, workplace size and time period (b-bottom graphs).

Fig. 1 shows prevalence of GHQ-12 caseness by motherhood and time-period (a-top graph) as well as motherhood, time period and workplace size (b-bottom graph) in our analytical sample. There is some indication of marked differences between working mothers and non-mothers in lockdown 3 (Fig. 1a) and this difference is apparent for those working in medium-sized enterprises.

To confirm these descriptive patterns, we ran mixed–effects logistic regressions. This allowed us to estimate odds and probabilities of psychological distress across our exposure groups. The results of the weighted, unadjusted Models 1 and 2 indicated no large effect differences with the adjusted models; however, there is a consistent pattern of negative confounding (i.e., odds in the adjusted models are higher) (Table 2).

**Fig. 2:**
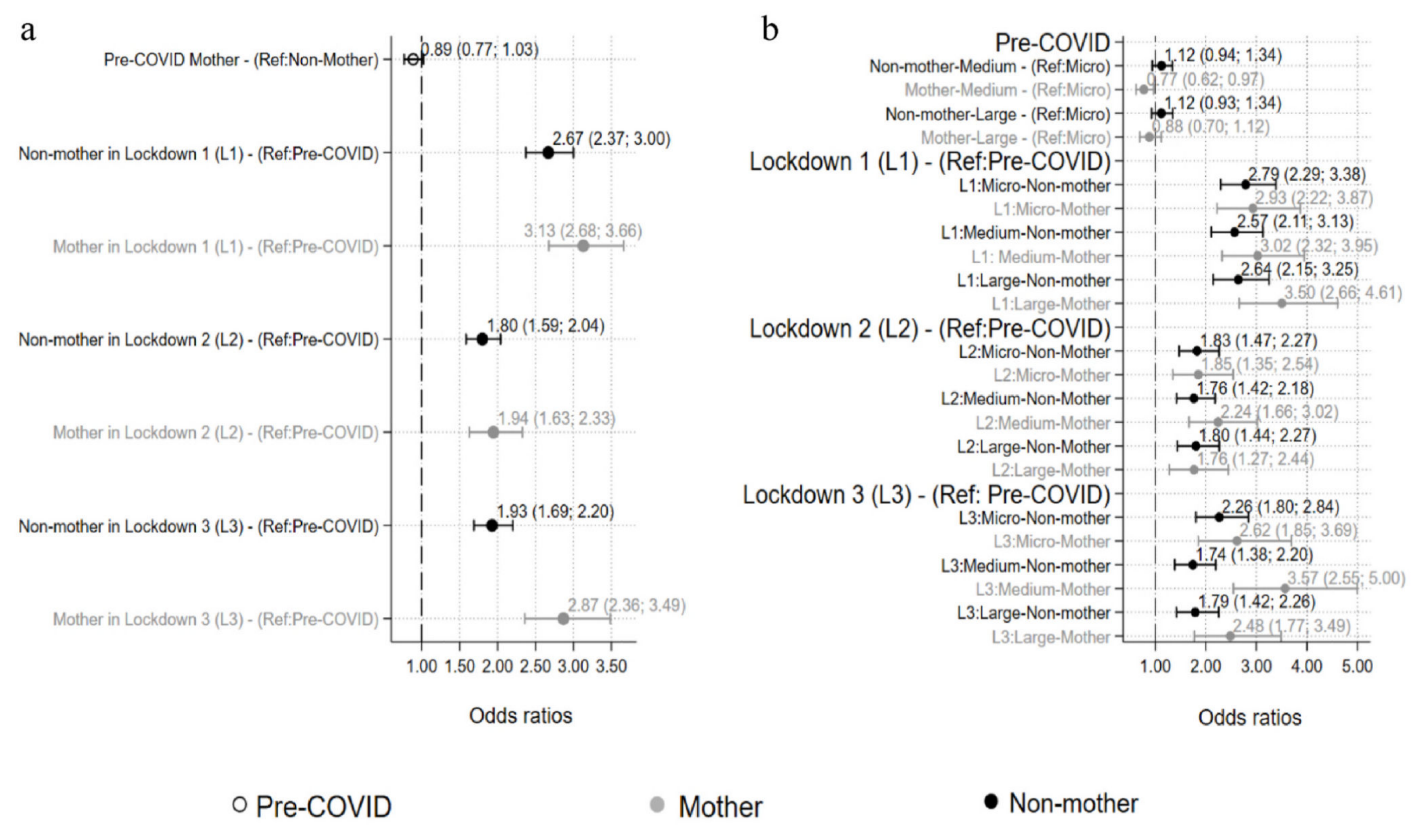
Adjusted OR for Model 1 (odds of having a GHQ-case by motherhood and time period and b) Adjusted OR for Model 2 (odds of having a GHQ-case by motherhood, time period and workplace size (L1: lockdown 1; L2: lockdown 2; and L3: lockdown 3).

In Model 1, when adjusted for all covariates (Table 2, Fig. 2a), odds of GHQ-12 caseness for working non-mothers were 2.7 times higher in the initial stage of lockdown 1 (OR: 2.67; 95% CI: 2.37 to 3.00) compared to the pre-COVID-19 period. In lockdown 2, the odds of GHQ-12 caseness for working non-mothers were 1.8 higher (OR: 1.80; 95% CI: 1.59 to 2.04) and in lockdown 3 the equivalent odds were 1.93 higher (OR: 1.93; 95% CI: 1.69 to 2.20). Working mothers showed similar trends with slighter higher odds for lockdowns 1 and 2 but marked differences for lockdown 3 (lockdown 1 OR: 3.13; 95% CI: 2.68 to 3.66; lockdown 2 OR: 1.94; 95% CI: 1.63 to 2.33; lockdown 3 OR: 2.87; 95% CI: 2.36 to 3.49). Adjustment produced an occurrence of negative confounding in both non-mothers and mothers indicating an underestimation in the unadjusted model. However, OR patterns did not change and still indicated higher odds of GHQ-12 caseness for working mothers in lockdown 3 compared to working non-mothers (Table 2).

In Model 2, we added workplace size to our exposures of Model 1 to check for moderation effects. As shown in Tables 2 and in the pre-COVID period there is no indication of differential effects in working women due to motherhood across workplaces of different sizes. GHQ-12 caseness for working non-mothers in medium size and large enterprises was not different from those working in micro enterprises (reference category). The same was observed for working mothers. Similar trends existed in the first two lockdown periods examined. While differences were observed in all groups compared to pre-COVID, there were no marked differences between working non-mothers and mothers in workplaces of different sizes. Also, in the first stages of lockdown 3 being a mother did not result in differential effects in GHQ-12 caseness for those working in micro and large enterprises; however, considerable differences were observed in the odds of GHQ-12 caseness for those working in medium-sized enterprises. In the latter case, the odds for working mothers were higher by more than two times compared to non-mothers (lockdown 3: mothers OR: 3.57, 95%CI:2.55-5.00; non-mothers OR: 1.74, 95%CI:1.38-2.20) (Fig. 2b).

**Fig. 3:**
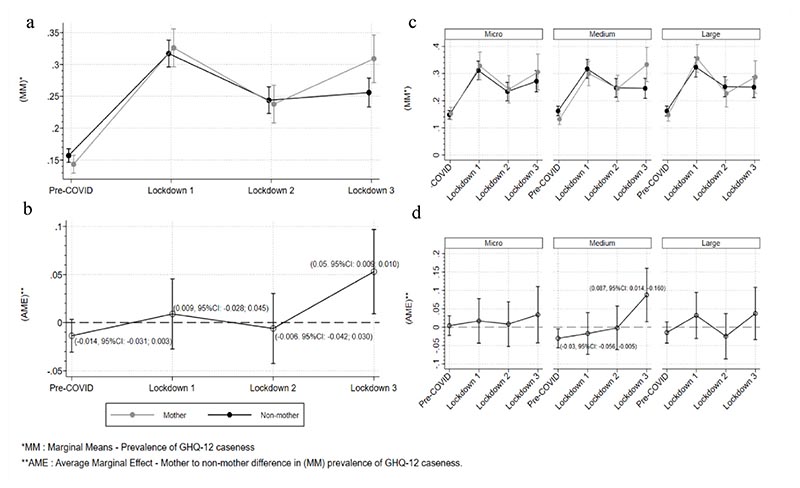
a) MM: Probability of a GHQ-case by time period (mothers and non-mothers) with 95% CIs, b) AME: Probability difference of a GHQ-case by time period (mothers Vs non-mothers) with 95% CIs, c) MM: Probability of a GHQ-case by time period and workplace size (mothers and non-mothers) with 95% CI, d) AME: Probability difference of a GHQ-case by time period and workplace size (mothers Vs non-mothers) with 95% CIs.

We also estimated marginal means (marginal probabilities) and average margins (differences between marginal probabilities) for both Models 1 and 2. Fig. 3a shows the probability of GHQ-12 caseness in all time periods for mothers and non-mothers. Fig. 3b shows the marginal probability differences between working mothers and non-mothers, where positive estimates indicate a higher probability of GHQ-12 caseness for working mothers. Fig. 3a indicates a difference in lockdown 3.

This is corroborated in Fig. 3b, demonstrating that there were marked differences in the prevalence of GHQ-12 caseness for working mothers in lockdown 3 compared to pre-COVID (lockdown 3: Average Margins (AM): 0.05; 95% CI: 0.009 to 0.097), but not in lockdowns 1 and 2 as the point estimates are very close resulting to both overlapping (a) and bidirectional (b) confidence intervals.

For Model 2 adjusted marginal means and average margins demonstrated marked differences of the estimated effect of motherhood on the prevalence of GHQ-12 caseness for those working in medium-sized enterprises in lockdown 3 compared to pre-COVID (lockdown 3: Medium sized: Average Margins (AM): 0.087; 95% CI: 0.014 to 0.160), compared to lockdowns 1 and 2 (Fig. 3c and d).

We also conducted two types of sensitivity analyses. First, those on furlough may have had different exposure profiles to SARS-CoV-2 infection risk (Oude Hengel et al., 2022), so we re-ran Models 1 and 2 analyses by excluding them from our analysis sample. No marked differences in the results were observed (Table A3). Moreover, we conducted sensitivity analysis using a lower cut off point for GHQ-case (3 ≤ GHQ-12 Case ≤12) and found no differences (Table A3).

## 5 Discussion

Overall, working women had an increased likelihood of psychological distress at the start of each of the three lockdowns compared to the pre-pandemic period. The lockdowns associated with the worst mental health were lockdowns 1 and 3. Our analysis did not reveal differences based on lockdown period, motherhood, and workplace size apart from lockdown 3 where working mothers were more likely to suffer from poorer mental health compared to working non-mothers. This was particularly the case for those working in medium-sized enterprises. Results were consistent in both relative (odds ratios) and additive (probabilities) terms.

Empirical literature related to COVID-19, shows that working women had a higher likelihood of experiencing psychological distress during the lockdown periods compared to the pre-COVID-19 period (Das et al., 2021; Hamouche, 2020; Kromydas et al., 2022; Magalhaes et al., 2021; Michael et al., 2022; C. L. Niedzwiedz et al., 2021b; Xiao et al., 2021). Our finding that working mothers were affected more than non-mothers not only aligns with this evidence but also expands our knowledge further on how mental health of working women specifically changed at the start of each UK lockdown. Our analyses showed that compared to before the onset of COVID-19 pandemic, working women were 2 times more likely to exhibit GHQ-12 caseness in lockdowns 1 & 3 and 1.2 times more likely in lockdown 2. This indicated that the risk of psychological distress increased when the pandemic first began (lockdown 1), slightly decreased in the 2nd lockdown, and rose again in the 3rd lockdown. These findings coincide with the pattern of lockdown stringency, lockdowns 1 and 3 had more restrictive measures in place compared to lockdown 2 (Baker et al., 2021; Michael et al., 2022; UK Government, 2020a, 2020b).

The study findings when employer size was included in our analyses demonstrated that odds of GHQ-12 caseness were higher for working mothers than working non-mothers only in lockdown 3. Thus, having children while working during the pandemic as a whole was not associated with higher deterioration in mental health in women in work; however, differential effects were identified only in lockdown 3. This aligns partially with research published in the US and in the global context that reported employees with children had an increased risk of stress during the pandemic as a result of increased childcare/home-schooling responsibilities (Hamouche, 2020; Michael et al., 2022; Zamarro and Prados, 2021). It is also in partial agreement with the study by Cheng et al. (2021) that found that during the first two months in the pandemic, working parents compared to non-parents were disproportionately impacted by the COVID-19 crisis. Our study goes beyond the first stage of the pandemic, to report how working mothers’ mental health evolved with each successive lockdown and we found that mothers and non-mothers were not differentially affected during the initial stages of lockdowns except in lockdown 3 and particularly for those working in medium-sized enterprises.

Examining the differences in restrictions between the three lockdowns, some potential reasons can provide partial explanation of our results (Fig. A1). Between lockdowns 1 and 2 there was a period (2–3 months) where most restrictions were lifted, and people had the chance to socialise and return to some of their usual activities, including leisure activities. Schools re-opened and remained open even during lockdown 2, before they closed again in lockdown 3. Moreover, in lockdown 2 people were encouraged to start going back to work, and the new Job Support scheme was in place, which was less generous and may have added more pressures on workplaces. These developments, separately or in combination(s), may have negatively affected the mental health of mothers more than non-mothers’ as childcare services were still very limited, and perhaps micro and medium-sized enterprises were still lacking the infrastructure for further supporting working from home (Ishimaru et al., 2021). This differential impact on mental health specifically for working women in medium size enterprises may be due to other reasons as well. While the majority of large enterprises offer occupational health support to their employees that face health issues and may need reasonable adjustments in the workplace, as well as proactive workplace health promotion interventions, the picture is vastly different in SMEs where less than a fifth of UK small employers have such provisions in place (Burge et al., 2023; Tu et al., 2019). The makeup of the workforce in micro, medium and large enterprises may explain this difference as well. Often, SMEs employ workers through agencies who may have less autonomy and control in their job, and this is more prevalent in medium size enterprises than micro and small enterprises (Cornick et al., 2017). Growth in the UK private business population over the last twenty years has mainly been due to increasing numbers of non-employing businesses, i.e. self-employed, who would have a greater degree of job control and autonomy over their work schedule and flexibility (BEIS, 2022). This fact compounding by our method of including those that reported they are self-employed in our micro group, may explain why working women in micro workplaces, despite having similar challenges in terms of resources and occupational health provision to medium size workplaces, did not show the same pattern. Furthermore, the types of jobs that are most prevalent in micro, medium and large workplaces may be another contributing factor as job type dictates to a large degree the demand, organisation of work, resources and rewards available. Wholesale and retail trade for example are the industrial sectors with the highest share in SMEs, whereas in large enterprises manufacturing has the highest share, followed by administrative and support services and wholesale and retail (BEIS, 2022). However, due to the small sample size it was not possible to investigate how specific occupations within our groupings may have contributed to the findings, specifically for medium size enterprises.

Lockdowns 1 and 3 were announced with no end date specified but for lockdown 2 an end date was clearly set and communicated. This may moderately explain the similarities in terms of the magnitude of the negative mental health effect between lockdowns 1 and 3 and also their difference to lockdown 2 equivalent effect.

This study advances our knowledge on how mental health disproportionately deteriorated following the COVID-19 pandemic outbreak (Almeida et al., 2020; Kromydas et al., 2022; Mutambudzi et al., 2020; C. L. Niedzwiedz et al., 2021b; Patel et al., 2022; Pieh et al., 2020; Pierce et al., 2020). Specifically, it examined the influence of the progression of the pandemic and variations in enforced restrictions on the mental health of working women. Demographics, family, economic and health related factors were adjusted for. This adjustment showed some negative confounding implying a rather underestimation of the unadjusted model. Our findings corroborate previous findings demonstrating that in the early stages of the pandemic, those who had young children at home had increased anxiety levels and were more likely to have psychological distress (Hampshire et al., 2021; Michael et al., 2022; Xiao et al., 2021). However, our results show that this disparity is persistent only in the initial stages of lockdown 3 and especially for mothers working in medium-sized enterprises.

There are several strengths to this study. It utilised Understanding Society’s nationally representative longitudinal dataset to analyse differences in GHQ-12 caseness across all three UK lockdowns in a systematic fashion. The analysis encompassed pre-COVID-19 outcome measures and three surveys of data collection after the start of the pandemic, enabling the research to investigate trends at each lockdown separately. It used precise, consistent methods for eligibility in the analytical sample and rigid time frames examining each lockdown consistently, which has not been done before. Previous studies have either used one lockdown, or a mix of lockdown and non-lockdown periods and subsequently over-generalize the effect of the pandemic and or lockdown periods. The richness of USoc’s database allowed for adjusting for multiple important covariates.

However, there are some limitations that should be noted. While using a nationally representative longitudinal dataset was a strength, at the same time it is a limitation as we cannot be certain how generalisable our findings are to other countries. Further macro-economic factors, such a labour market, government support to workplaces during the pandemic, severity of restrictions, available benefits, as well as the variable healthcare provisions across countries are some factors that may impact generalisability. In terms of our outcome, only one mental health measure (GHQ-12 caseness) was included, and the results may vary if other measures of anxiety and/or depression were used. Non-response bias and to some extent recall bias cannot be dismissed (Kromydas et al., 2022). Moreover, due to focusing only on working women using a survey that is designed to be representative on the whole population, sample representativeness is not guaranteed. Even though USoc is representative of the whole population, we do not know whether this holds when analysis is confined to only a specific subsample. Therefore, we were not able to use USoc’s sampling weights because they were constructed using a number of variables, which values might differ considerably between genders. However, we calculated weights for outcome missingness as well as tested for covariate proportional imbalances between pre-COVID and each lockdown survey. We found that our sample was balanced with only minor proportional differences. Moreover, the methodology used to record the values for employer size in the original USoc variable does not allow for representation of the traditionally used definition of Small and Medium Enterprises (EUR-Lex, 2003). Moreover, we used a model whose structure implies that observations are clustered within individuals and the number of observations within each cluster is limited. As shown in Table A2 the minimum number of observations within a cluster is 2 (respondents participated in at least one pre-pandemic wave and in at least one lockdown), and the maximum is 6 (participated in all pre-pandemic and pandemic waves). It is true that the greater the cluster size the more powerful the model is in terms of estimating random effects precisely (Austin and Leckie, 2018), however there are a number of studies arguing that small cluster sizes are unlikely to cause serious bias in estimations (Bell et al., 2008; P. Clarke, 2008; Philippa Clarke and Wheaton, 2007; Maas and Hox, 2005).

It is important to recognize as well that the context of the COVID-19 crisis potentially influenced participant response more broadly (Kromydas et al., 2022). Furthermore, inconsistencies in variables and their availability meant that covariates that could be important, including homeworking, occupation and/or industry membership, were excluded. Specifically for homeworking, exclusion was based on two reasons. First, missing values in this variable reduced our sample by almost 30% and given our aforementioned limitations on representability and restrictions on sampling weighting, the inclusion of this variable would seriously threaten the generalisability of our findings. Secondly, homeworking prevalence increased abruptly during pandemic and was applied to all except the key workers. This contrasts with pre-COVID homeworking, which was considered a work-benefit and was relatively rare and applicable mainly to specific industries and occupations (Reuschke, 2019; Wheatley, 2016). Furlough was specific to the pandemic, and we therefore had no data to compare to pre-COVID, but we partially accounted for this by conducting sensitivity analysis excluding those participants in furlough and re-run our analysis.

The findings have important implications for workplace, mental, female, and public health. In the current study, we aimed to address other determinants that were lacking in previous studies, by conducting analysis by motherhood and workplace size. Our results did not show big differences in lockdowns 1 and 2, but they call attention to the disparities in GHQ-12 caseness between working mothers and working non-mothers as well as by employer size during lockdown 3, where mothers, and particularly those working in medium-sized companies were affected considerably more. Plausible explanations could relate to both the type of restrictions applied or the fact that the third lockdown was essentially a continuation of the second one, as well as the known seasonal mental health effect since lockdown 1 happened during summer and lockdown 2 and 3 during the winter (Banks and Xu, 2020; Michael et al., 2022). This emphasises not only the mental health afflictions related to the pandemic as the whole but the heterogeneity of the effect in each of the three lockdowns as well as the complexity of the intersection of motherhood and nature of workplace, one component of which is its size.

Working women, especially those with children in their household, have fewer safety nets and may experience greater strain due to increased childcare responsibilities while employed during the pandemic (Carroll et al., 2020; Dollberg et al., 2021; Elliott et al., 2021; Michael et al., 2022). Employers of medium and small size often have less resources and systems in place to support staff and staff absences which can result in more pressure/stress in employees (Bradshaw et al., 2001a; B. Nowrouzi et al., 2016a). Post-pandemic policies and interventions are necessary to support the mental health of working women (Michael et al., 2022), and support medium and small size employers (BMA, 2022; Nishikido et al., 2023). Our recommendation is timely as the UK government acknowledged the disproportionate negative mental health effect of COVID-19 pandemic on women in their 2023 Spring Budget. On top of this, in January 2023 it also announced a fund of £1 million to boost health at work (HM Treasury, 2023). Further research is needed to understand the mechanisms that drive these findings with respect to lockdowns and periods of high work and family strain. In conjunction with declines in psychological well-being, long waiting times to receive healthcare persist. Therefore, evaluating the mental health of disproportionately affected populations can inform both non-workplace and workplace interventions to steer already limited resources to those with the greatest need (Czabala et al., 2011; Kallitsoglou and Topalli, 2021; S. L. Wagner et al., 2016). Policies unique to these exposures are required to reduce the widened gendered health inequality precipitated by the pandemic and longer-term longitudinal studies are needed to understand potential lasting effects. The inclusion of female’s knowledge and experience is necessary for creating post-COVID-19 recovery interventions and pandemic crisis management (Kallitsoglou and Topalli, 2021; Power, 2020; Wenham et al., 2020).

## Supplementary Material

Supplementary Material

## Figures and Tables

**Table 1:** Descriptive statistics for outcome, exposure and other covariates used for model adjustment by time period (counts and percentages).

	Counts					Percentages (%)				
	Pre-COVID	Lockdown 1	Lockdown 2	Lockdown 3	Total	Pre-COVID	Lockdown 1	Lockdown 2	Lockdown 3	Total
***GHQ-case***										
No case	11,272	3201	2549	2076	19,098	78.22	63.69	70.69	68.42	73.24
Case	3139	1825	1057	958	6979	21.78	36.31	29.31	31.58	26.76
***Motherhood***										
No mother	9366	3310	2495	2153	17,324	64.99	65.86	69.19	70.96	66.43
Mother	5045	1716	1111	881	8753	35.01	34.14	30.81	29.04	33.57
***Workplace size***										
1-24	5106	1693	1234	1001	9034	35.43	33.68	34.22	32.99	34.64
25-199	5031	1770	1245	1060	9106	34.91	35.22	34.53	34.94	34.92
>200	4274	1563	1127	973	7937	29.66	31.10	31.25	32.07	30.44
***Ethnicity***										
White	12,900	4510	3248	2759	23,417	89.51	89.73	90.07	90.94	89.8
Non-white	1511	516	358	275	2660	10.49	10.27	9.93	9.06	10.20
***Cohabitation***										
No	4318	1443	1126	940	7827	29.96	28.71	31.23	30.98	30.01
Yes	10,093	3583	2480	2094	18,250	70.04	71.29	68.77	69.02	69.99
***UK countries***										
England	11,684	4064	2956	2493	21,197	81.08	80.86	81.97	82.17	81.29
Wales	849	293	208	161	1511	5.89	5.83	5.77	5.31	5.79
Scotland	1252	456	303	260	2271	8.69	9.07	8.40	8.57	8.71
N. Ireland	626	213	139	120	1098	4.34	4.24	3.85	3.96	4.21
***Qualifications***										
Tertiary	8372	2946	2129	1805	15,252	58.09	58.62	59.04	59.49	58.49
Secondary	5214	1804	1292	1085	9395	36.18	35.89	35.83	35.76	36.03
None	825	276	185	144	1430	5.72	5.49	5.13	4.75	5.48
***Working hours***										
0-15	1717	1968	778	728	5191	11.91	39.16	21.58	23.99	19.91
16-35	6696	1455	1366	1075	10,592	46.46	28.95	37.88	35.43	40.62
>36	5998	1603	1462	1231	10,294	41.62	31.89	40.54	40.57	39.48
***Financial situation***									
Good	10,590	3937	2766	2352	19,645	73.49	78.33	76.71	77.52	75.33
Bad	3821	1089	840	682	6432	26.51	21.67	23.29	22.48	24.67
***Baseline health***										
no	10,480	3624	2570	2174	18,848	72.72	72.11	71.27	71.65	72.28
yes	3931	1402	1036	860	7229	27.28	27.89	28.73	28.35	27.72
**Age (cont.)**	**mean**					**Standard deviation**			
	44.52*	45.37	47.10	47.51	45.39	12.21	12.43	12.20	12.15	12.30
**Total**	**14,411**	**5026**	**3606**	**3034**	**26,077**	**100.00**	**100.00**	**100.00**	**100.00**	**100.00**

Note: * (average age and standard deviation for all within the pre-pandemic period: Wave 9: 43.98 (sd:11.90); Wave 10: 44.53 (sd: 12.24); Wave 11: 45.02 (sd:12.45).

**Table 2:** Estimates in terms of Odds Ratios (OR) for Model 1 (two-way interaction between motherhood and time) and Model 2 (three-way interaction among motherhood, workplace size and time).

	Unadjusted						Adjusted					
	Non-mother			Mother			Non-mother			Mother		
	Est	95%CI		Est	95%CI		Est	95%CI		Est	95%CI	
	**OR**	**Lower**	**Higher**	**OR**	**Lower**	**Higher**	**OR**	**Lower**	**Higher**	**OR**	**Lower**	**Higher**
**Model 1**												
**Pre-COVID**				0.98	0.86	1.12				0.89	0.77	1.03
**Lockdown 1 (L1) (Ref: Pre-COVID)**	2.61	2.33	2.92	3.05	2.61	3.55	2.67	2.37	3.00	3.13	2.68	3.66
**Lockdown 2 (L2) (Ref: Pre-COVID)**	1.70	1.50	1.93	1.84	1.54	2.21	1.80	1.59	2.04	1.94	1.63	2.33
**Lockdown 3 (L3) (Ref: Pre-COVID)**	1.81	1.59	2.07	2.70	2.22	3.28	1.93	1.69	2.20	2.87	2.36	3.49
**Model 2**												
**Pre-COVID-Medium (Ref: Pre-COVID-Micro)**	1.14	0.95	1.37	0.86	0.69	1.07	1.12	0.94	1.34	0.77	0.62	0.97
**Pre-COVID-Large (Ref: Pre-COVID-Micro)**	1.13	0.93	1.36	0.94	0.74	1.19	1.12	0.93	1.34	0.88	0.70	1.12
**L1-Micro (Ref: Pre-COVID-Micro)**	2.83	2.34	3.43	3.02	2.30	3.97	2.79	2.29	3.38	2.93	2.22	3.87
**L1-Medium (Ref: Pre-COVID-Medium)**	2.49	2.05	3.02	2.92	2.24	3.82	2.57	2.11	3.13	3.02	2.32	3.95
**L1-Large (Ref: Pre-COVID-Large)**	2.50	2.03	3.07	3.25	2.46	4.31	2.64	2.15	3.25	3.50	2.66	4.61
**L2-Micro (Ref: Pre-COVID-Micro)**	1.75	1.41	2.17	1.83	1.33	2.53	1.83	1.47	2.27	1.85	1.35	2.54
**L2-Medium (Ref: Pre-COVID-Medium)**	1.68	1.35	2.08	2.10	1.55	2.83	1.76	1.42	2.18	2.24	1.66	3.02
**L2-Large (Ref: Pre-COVID-Large)**	1.66	1.32	2.08	1.63	1.17	2.26	1.80	1.44	2.27	1.76	1.27	2.44
**L3-Micro (Ref: Pre-COVID-Micro)**	2.17	1.73	2.72	2.57	1.82	3.62	2.26	1.80	2.84	2.62	1.85	3.69
**L3-Medium (Ref: Pre-COVID-Medium)**	1.64	1.31	2.07	3.35	2.39	4.68	1.74	1.38	2.20	3.57	2.55	5.00
**L3-Large (Ref: Pre-COVID-Large)**	1.64	1.30	2.07	2.26	1.61	3.17	1.79	1.42	2.26	2.48	1.77	3.49

## Data Availability

Data will be made available on request.
