# Supplementary material for: COVID-19 lockdowns and working women’s mental health: Does motherhood and size of workplace matter? A comparative analysis using understanding society
Source: Soc Sci Med. Author manuscript; Available in PMC 2023 Dec 1. (PMC7615337; doi:10.1016/j.socscimed.2023.116418)
Supplement: Supplementary Material [file EMS191745-supplement-Supplementary_Material.pdf]

SUPPLEMENTARY MATERIAL

Appendix A

Figure A1. The 3 UK lockdowns and their restrictions (Baker et al., 2021; UK Government, 2020; UK government, 2020; UK Government, 2021)

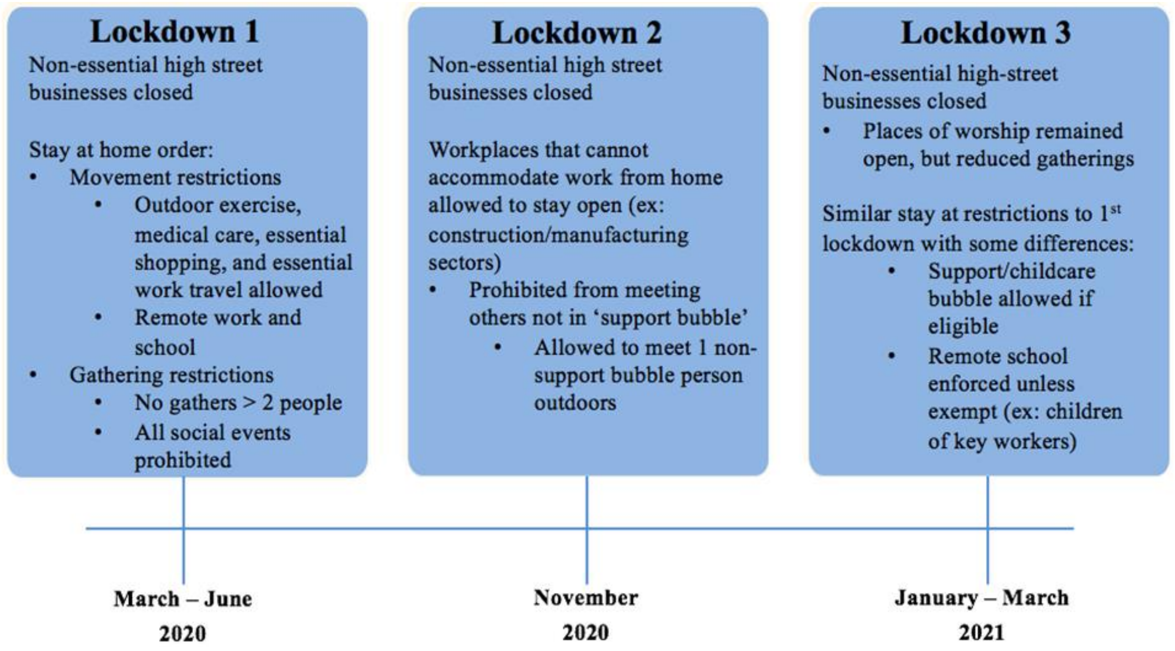

Figure A2. Flowchart of study participants

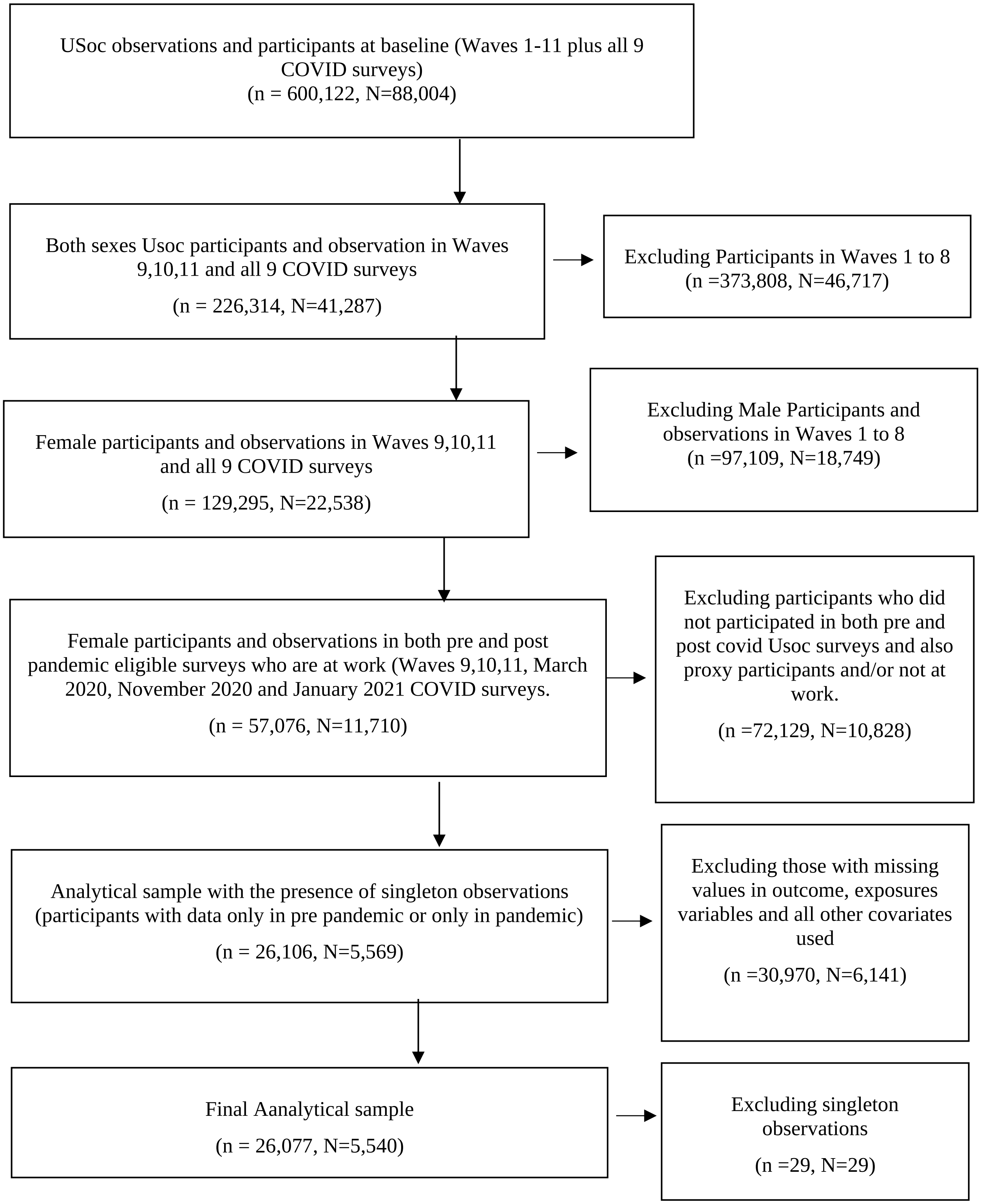

Methods

Full-time work hours were categorised as working greater than or equal to 35 hours per week, per the UK government's classification (UK Government, n.d.). In the UK, a Small and Medium Enterprise (SME) is defined as an enterprise with less than 250 employees (UK Government, n.d.). However, the answer options for USoc's variables that represent enterprise size do not align with this definition as it has values for employer size between 100-199 or 200-499 (University of Essex, Institute for Social and Economic Research, 2021; University of Essex, Institute for Social and Economic Research, 2022). Therefore, for the purposes of this research, an SME was classified as 200 employees or less.

A few of the aforementioned confounders were time-invariant; and therefore, were not asked consistently in each wave of interest, subsequently yielding missing observations. The time-invariant variables were industry, enterprise size, and level of educational qualification. Where observations were missing for these confounders, data were carried forward from a prior, relevant wave(s). The job industry variable was not asked in COVID-19 wave 1 or 6. Therefore, for wave 1, observations were fed-forward from the merged mainstage survey waves 9-11. For wave 6, observations were fed-forward from COVID-19 waves 3 and 4. Enterprise size was not asked about in COVID-19 wave 1 or 6 either. For this variable in these waves, observations were fed-forward from the merged mainstage survey waves 9-11. The level of educational qualification variable was not asked in any of the COVID-19 waves of interest; therefore, observations for this variable were fed-forward from merged mainstage survey waves 9-11 for COVID-19 waves 1, 6, and 7. This method of accounting for missing data and time-invariant variables was adopted from Kromydas et al (2022) research methods. Furthermore, it was justifiable to do so because a change in response for the time-invariant variables was unlikely to happen within lockdowns given that the descriptive statistics associated with the consistently asked variables did not fluctuate substantially across time periods (see Table 2).

Weighting

We calculated inverse probability weights based on age (Young:16/49, Old: Older than 50), ethnicity (White, Non-white), UK country of residence (England, Wales, Scotland, Northern Ireland) and qualification level (None or Low, Intermediate, Higher) within each wave to account for missing data in our outcome variable[1].

Table A1: Participation times by respondent and corresponding observations

Participation Times	No of Respondents	Percent	No of Observations	Percent
2	278	5.0	556	2.1
3	651	11.8	1,953	7.5
4	1,529	27.6	6,105	23.4
5	998	18.0	4,959	19.0
6	2,084	37.6	12,504	48.0
**Total**	**5,540**	**100.0**	**26,077**	**100.0**

[1] Seaman SR, White IR. Review of inverse probability weighting for dealing with missing data. Statistical methods in medical research. 2013 Jun;22(3):278-95.

Table A2. Transition probabilities for variable on workplace size (One wave and two waves difference)

	One wave difference				Two waves difference			
**Workplace size**	**1 to 25 employees**	**25 to 199 employees**	**Over 200 employees**	Total	**1 to 25 employees**	**25 to 199 employees**	**Over 200 employees**	**Total**
**1 to 25 employees**	**93.37**	4.68	1.95	100.00	**93.36**	4.71	1.93	**100.00**
**25 to 199 employees**	5.60	**90.24**	4.16	100.00	5.70	**90.14**	4.16	**100.00**
**Over 200 employees**	2.58	3.73	**93.69**	100.00	2.61	3.85	**93.54**	**100.00**
**Total**	**40.69**	**31.44**	**27.88**	100.00	**40.58**	**31.46**	**27.96**	**100.00**

Sensitivity Analysis

Table A3: Sensitivity Analyses

Model 1	Sensitivity 1						Sensitivity 2					
	Non-mother			Mother			Non-mother			Mother		
	Est	95%CI		Est	95%CI		Est	95%CI		Est	95%CI	
**Model 1**	**OR**	**Lower**	**Higher**	**OR**	**Lower**	**Higher**	**OR**	**Lower**	**Higher**	**OR**	**Lower**	**Higher**
**Pre-COVID**				0.91	0.78	1.06				0.90	0.78	1.03
**Lockdown 1 (L1) (Ref: Pre-COVID)**	2.64	2.33	2.99	3.29	2.79	3.89	3.24	2.90	3.62	3.79	3.26	4.40
**Lockdown 2 (L2) (Ref: Pre-COVID)**	1.76	1.53	2.02	2.07	1.70	2.51	1.84	1.63	2.08	1.88	1.58	2.24
**Lockdown 3 (L3) (Ref: Pre-COVID)**	1.96	1.69	2.27	2.78	2.24	3.45	2.01	1.77	2.29	2.78	2.30	3.36
**Model 2**												
**Pre-COVID-Medium (Ref: Pre-COVID-Micro)**	1.16	0.96	1.41	0.79	0.62	1.00	1.14	0.97	1.35	0.73	0.59	0.90
**Pre-COVID-Large (Ref: Pre-COVID-Micro)**	1.16	0.95	1.42	0.91	0.71	1.16	1.10	0.92	1.31	0.95	0.76	1.18
**L1-Micro (Ref: Pre-COVID-Micro)**	2.86	2.30	3.55	3.16	2.31	4.31	3.59	2.97	4.33	3.42	2.61	4.49
**L1-Medium (Ref: Pre-COVID-Medium)**	2.86	2.27	3.61	3.22	2.44	4.25	3.23	2.64	3.96	4.16	3.24	5.33
**L1-Large (Ref: Pre-COVID-Large)**	3.03	2.40	3.84	3.53	2.67	4.68	3.69	2.99	4.55	3.82	2.94	4.96
**L2-Micro (Ref: Pre-COVID-Micro)**	1.74	1.34	2.24	1.79	1.23	2.60	1.88	1.53	2.31	1.78	1.31	2.42
**L2-Medium (Ref: Pre-COVID-Medium)**	1.93	1.49	2.50	2.54	1.86	3.48	2.03	1.63	2.53	2.26	1.69	3.03
**L2-Large (Ref: Pre-COVID-Large)**	2.15	1.67	2.77	1.90	1.36	2.65	2.06	1.63	2.60	1.63	1.19	2.24
**L3-Micro (Ref: Pre-COVID-Micro)**	2.23	1.71	2.81	2.40	1.56	3.68	2.28	1.83	2.83	2.46	1.74	3.49
**L3-Medium (Ref: Pre-COVID-Medium)**	2.05	1.56	2.69	3.86	2.68	5.56	2.08	1.65	2.62	3.48	2.52	4.80
**L3-Large (Ref: Pre-COVID-Large)**	2.22	1.70	2.89	2.25	1.58	3.20	2.15	1.69	2.74	2.46	1.79	3.39

*OR: Odds Ratios
